# The Potential of Antiviral Peptides as COVID-19 Therapeutics

**DOI:** 10.3389/fphar.2020.575444

**Published:** 2020-09-15

**Authors:** Arun Suria Karnan Mahendran, Yin Sze Lim, Chee-Mun Fang, Hwei-San Loh, Cheng Foh Le

**Affiliations:** ^1^ School of Biosciences, Faculty of Science and Engineering, University of Nottingham Malaysia, Semenyih, Malaysia; ^2^ Division of Biomedical Sciences, School of Pharmacy, Faculty of Science and Engineering, University of Nottingham Malaysia, Semenyih, Malaysia

**Keywords:** antiviral peptides, antimicrobial peptides, antiviral prophylaxis, antiviral agents, COVID-19, SARS-CoV-2, coronaviruses, infectious disease****

## Introduction

To date, more than 17 million cases of coronavirus disease 2019 (COVID-19) and 900,000 deaths have been reported globally (World Health Organization, 2020). Major collaborative efforts focusing on the development of anti-SARS-CoV-2 vaccines and antiviral therapeutics are accelerated at an unprecedented rate. Various therapeutic agents quickly taken into clinical trials are largely based on existing drugs with non-specific antiviral activities or compounds pharmacologically speculated to be effective in enhancing the overall clinical outcome of COVID-19 patients. To name a few, these include the nucleoside inhibitor prodrug remdesivir (GS-5734), the antiparasitic drug ivermectin, the HIV protease inhibitor nelfinavir, the anti-inflammatory drug cepharanthine, the antimalarial drug hydroxychloroquine, the general steroid dexamethasone, and others (Caly et al., 2020; Ledford, 2020; Ohashi et al., 2020; Vanden Eynde, 2020; Wang Y. et al., 2020). However, only dexamethasone and remdesivir appear to confer promising treatment outcomes by reducing deaths or time to clinical improvement in the severe COVID-19 patients (Grein et al., 2020; Ledford, 2020; Wang Y. et al., 2020). The world is extremely desperate for a cure or prophylaxis against SARS-CoV-2 with the hope of saving more lives. Nevertheless, little has been described for antiviral peptides (AVPs) or alternatively known as antimicrobial peptides (AMPs) that possess antiviral activities. AVPs are a class of short (8–40 amino acids in length) polycationic antivirals with potent broad spectrum antiviral activities (Chang and Yang, 2013; Skalickova et al., 2015; Ahmed et al., 2019; Nyanguile, 2019; Sala et al., 2019; Vilas Boas et al., 2019). Interestingly, there are AVPs demonstrated to exert prophylactic and therapeutic effects against coronaviruses (CoVs). In this opinion paper, we describe the potential use of AVPs against COVID-19 based on the documented evidence against SARS-CoV2, SARS-CoV, MERS-CoV, SARS-related CoVs, and other respiratory viruses that shall warrant further development of this class of compounds in the face of the current pandemic threat.

## Mechanism of Viral Infection

SARS-CoV-2 is an enveloped virus with the characteristic spike (S) glycoprotein to mediate viral entry *via* the cell surface receptor angiotensin-converting enzyme-2 (ACE2) (Zhou et al., 2020). The S1 subunit of S protein is the receptor-binding domain responsible for ACE2 binding (Xia et al., 2020b). Subsequently, a cellular serine protease TMPRSS2 is required for S protein priming, which induces proteolytic cleavage of S protein at S1/S2 and S2’ sites (Hoffmann et al., 2020). Following cleavage, both heptapeptide repeat 1 (HR1) and heptapeptide repeat 2 (HR2) regions of the S2 subunit of S protein interact to form the 6-helix bundle (6HB) fusion core (Liu et al., 2004; Kang et al., 2020). The formation of 6HB is critical in facilitating the viral membrane fusion process for viral entry into the host cell *via* endocytosis. During the late endosomal stage, endosomal acidification would induce virus-endosome membrane fusion, thereby leading to viral uncoating (viral RNA release) for the initiation of viral replication and infection (Du et al., 2009; Das et al., 2010; Letko et al., 2020). Notably, the S1 subunit of SARS-CoV-2 showed a 10- to 20-fold higher ACE2 binding affinity comparing to SARS-CoV, which may explain the higher transmissibility and infectivity of COVID-19 (Wrapp et al., 2020).

## Targeting the Viral Envelope

**Figure 1:**
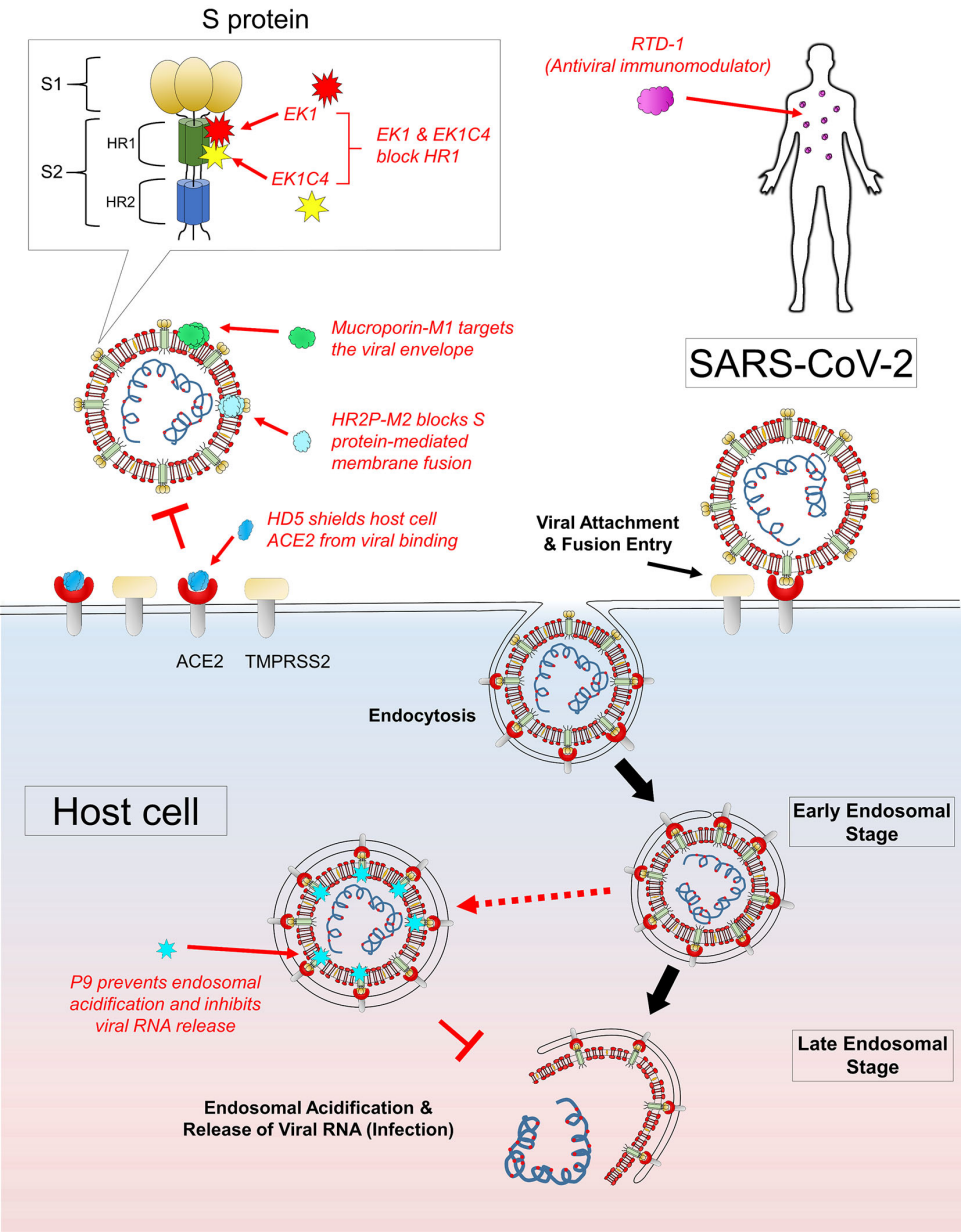
Mechanism of actions of AVPs with potential anti-SARS-CoV-2 activities. Several AVPs target the key structural components of the virus to exert antiviral effects: mucroporin-M1 acts by disrupting viral envelope, HR2P-M2 targets the viral S protein-mediated fusion, EK1 and EK1C4 block the HR1 domain of viral S2 subunit, and P9 peptide inhibits late endosomal acidification and thus preventing viral RNA release. There are AVPs which confer antiviral protection to the host: RTD-1 is a potent antiviral immunomodulator to trigger protective immunity, and HD5 binds to and shields ACE2 from viral recognition and binding.

Mucroporin-M1 (LFRLIKSLIKRLVSAFK) is a peptide analog designed with four residual mutations at positions G3R, P6K, G10K, and G11R from the parent peptide mucroporin (LFGLIPSLIGGLVSAFK) isolated from the venom of the scorpion *Lychas mucronatus* (Dai et al., 2008; Li et al., 2011). Such increase in cationicity enhanced the dipolar characteristic of the peptide helix, which contributed to the drastic increment in antiviral activity against SARS-CoV (50% virus infection, EC_50_ of 14.46 µg/ml), influenza H5N1 (EC_50_ of 2.10 µg/ml), and measles (EC_50_ of 7.15 µg/ml) pseudoviruses (Li et al., 2011). Based on the observations that mucroporin-M1 directly interacted with measles viral particles and a greater antiviral effect with pre-treatment, mucroporin-M1 was proposed to serve the role of a molecular blocker, which must find its target before viral attachment to the host cells (**Figure 1**). Following peptide-virus binding, the strong electrostatic affinity of mucroporin-M1 could allow interaction and disruption of the viral envelope, thereby exerting a direct virucidal effect against SARS-CoV, MERS-CoV, and influenza H5N1 viruses (Li et al., 2011).

## Targeting the Spike (S) Glycoprotein

EK1 (SLDQINVTFLDLEYEMKKLEEAIKKLEESYIDLKEL) is a pan-CoV fusion inhibitor designed by Xia and colleagues. This 36-residue peptide showed high cross-reactivity against all SARS-CoV, MERS-CoV, and three SARS-related CoVs from bats tested (Xia et al., 2019). As shown in **Figure 1**, EK1 acts by blocking the HR1 domain to disrupt the formation of the 6HB core, which causes inhibition of viral fusion entry into the host cell (Xia et al., 2020b). Intranasal application of EK1 further protected mice from pre- and post-challenges by the HCoV-OC43 alphacoronavirus and MERS-CoV challenges. Such dual therapeutic and prophylactic effects displayed by a single compound against various closely related CoVs are highly desirable. The broad spectrum anti-CoV activity strongly suggests that EK1 could be useful against SARS-CoV-2 infection. Recently, a highly potent lipopeptide variant EK1C4 was generated by C-terminally conjugating EK1 with a cholesterol moiety using a glycine/serine linker and a polyethylene glycol (PEG) spacer (-EK1-GSGSG-PEG4-chol) (Xia et al., 2019). The results were exciting, where SARS-CoV-2 cell-cell fusion was severely impaired at 50% inhibitory concentration (IC_50_) of 1.3nM, while anti-SARS-CoV-2 pseudoviruses infection activity was recorded at 15.8 nM. Notably, the SARS-CoV-2 HR1 protein binding affinity of EK1C4 was drastically enhanced by 226-fold, which also explained the observed 149-fold higher in antiviral activity of EK1C4 as compared to EK1. It was hypothesized that the conjugated cholesterol might have a profound role in facilitating EK1C4 binding toward the HR1 protein. Indeed, cholesterol conjugation enhancement of antiviral activity had previously been reported with the HIV-1 fusion inhibitor C34 (Hollmann et al., 2013). Besides EK1C4, HR2P-M2 was a peptide designed based on the 6HB core of MERS-CoV HR1 and HR2 domains (Channappanavar et al., 2015). This peptide acted by blocking the S protein-mediated membrane fusion mechanism of MERS-CoV. Of note, intranasal administration of HR2P-M2 was found to significantly reduce lung viral titers by >1,000-fold and protected the mice from MERS-CoV infection (Channappanavar et al., 2015). However, the narrow specificity of HR2P-M2 against MERS-CoV but not SARS-CoV may indirectly suggest an insufficient cross-reactivity with HR2P-M2 against other betacoronaviruses.

## Inhibition of Endosomal Acidification

The mouse β-defensins-4 derived P9 (NGAICWGPCPTAFRQIGNCGHFKVRCCKIR) was shown to bind to the MERS-CoV S2 subunit and remained co-localized with the viruses without inhibiting viral entry into the host cell *via* endocytosis (Zhao et al., 2016). While in the endosomes, the polycationic property of P9 induced a basic microenvironment to prevent endosomal acidification of the late endosomes. Without endosomal acidification, the pH-dependent activation of viral fusion proteins to initiate viral-host endosomal membrane fusion failed to occur and thus the critical step in viral uncoating prior to viral RNA release (**Figure 1**). This mechanism of action was indeed highly effective, broadly inhibiting SARS-CoV, MERS-CoV, and a diverse panel of influenza viruses H1N1, H3N2, H5N1, H7N7, and H7N9. Such target specificity nature of P9 also partly explained the low cytotoxic property of P9 (IC_50_ of 380 µg/ml) against the mammalian Madin-Darby canine kidney cells tested. The broad therapeutic windows of P9 would be a significant advantage in the future development of P9 as an antiviral agent. Notably, P9 displayed both prophylactic and therapeutic effects as observed in an *in vivo* SARS-CoV mouse model of infection (Zhao et al., 2016).

## For the Host: Shielding the Cell Receptors

ACE2 is well-known as the primary receptor for CoV at the first step of viral infection. It was found that HD5 (ATCYCRTGRCATRESLSGVCEISGRLYRLCCR), a natural lectin-like human defensins-5 (HD5) peptide secreted by the Paneth cells in the crypts of Lieberkuhn, could interact with glycosylated proteins and lipid components (Wang C. et al., 2020). Based on the structural and biochemical properties, HD5 was initially hypothesized to recognize and inhibit SARS-CoV-2 S protein, host cell ACE2 or both of these target components (Wang C. et al., 2020). However, it was later confirmed that HD5 competitively blocked ACE2 receptors on the host cells instead of targeting the viral S1 subunit (**Figure 1**). The high-affinity binding between HD5 and the ligand-binding domain of ACE2 through the formation of multiple hydrogen bonds effectively shielded (protected) the host cells from viral recognition and infection.

## Antiviral Immunomodulatory Effect

The cyclic peptide RTD-1 (GFCRCLCRRGVCRCICTR) from rhesus macaque leukocytes was reported to decrease disease pathogenesis of SARS-CoV infection in mice, as observed with a substantial reduction in perivascular infiltrate and necrotizing bronchiolitis (Wohlford-Lenane et al., 2009). Interestingly, neither did RTD-1 inhibit the virus or interact with the host cell receptors to exert an antiviral effect. In contrast, it was noticed that the virus titers and lung tissue nucleocapsid (N) gene antigen expression were similar to the untreated control mice. Together with an increase in cytokine levels of interleukin-6, keratinocyte chemoattractant, and granulocyte colony-stimulating factor in lung cell homogenates, RTD-1 was suggested to act as an immunomodulatory effector molecule *via* a blunted proinflammatory cytokine response in eliminating SARS-CoV (Wohlford-Lenane et al., 2009).

## Discussion

Prioritization and emergency preparedness in responding to the emerging infectious diseases associated with outbreaks and pandemics are of utmost urgency. In search of an effective antiviral against COVID-19, we believe AVPs could represent one of the potential classes of new antiviral agents against SARS-CoV-2. It is fascinating to discover how AVPs, composed principally of short and simple amino acid sequences, could interact with and specifically target the different viral components to achieve potent antiviral effects. Here, we would like to bring the attention to a number of AVPs with highly promising anti-CoV activities: mucroporin-M1 disrupts viral envelope, HR2P-M2 targets the viral S protein-mediated fusion mechanism, EK1 and EK1C4 block the HR1 domain of viral S2 subunit, and P9 peptide inhibits late endosomal acidification and thus preventing viral RNA release. Besides, there are AVPs that confer protection to the host: RTD-1 is an antiviral immunomodulator that triggers protective immunity; HD5 binds to and shields host ACE2 receptor to prevent viral recognition and attachment. Recent findings have gathered evidence on the potential role of a host serine protease TMPRSS2 in facilitating the S protein priming process of SARS-CoV-2 that is crucial for the subsequent viral membrane fusion events (Hoffmann et al., 2020). Furthermore, the newly discovered S protein furin-like cleavage site and the novel CD147-mediated viral entry pathway could have important implications in SARS-CoV-2 viral pathogenicity (Coutard et al., 2020; Wang K. et al., 2020; Xia et al., 2020a). These key viral components could represent valuable targets in the development of novel antiviral agents. Cocktail therapy using a selected combination of AVPs or as supplemental therapeutics in combination with other classes of antiviral agents could be a promising treatment strategy that would worth further clinical investigations (Vilas Boas et al., 2019). Such optimism is not unfounded, as enfuvirtide, a 36-amino acid fusion inhibitor AVP, was approved by the US Food and Drug Administration in 2003 for treatment against human immunodeficiency virus in combination with other antiretroviral drugs (Lalezari et al., 2003; Fung and Guo, 2004; Poveda et al., 2005). It acts by blocking the HR1 domain of the viral envelope glycoprotein 41. Sifuvertide, an AVP with enhanced potency and a lower threshold for resistance than enfuvirtide, is currently under phase III trials in China (He et al., 2008; Wang et al., 2009; Su et al., 2015; Yu et al., 2018). Although clinical trials of AVPs against CoVs have not been reported thus far, the dire need for an effective anti-SARS-CoV-2 agent could potentially reshape the drug discovery landscape as with the initiation of Operation Warp Speed by the US government in April 2020 for COVID-19 vaccine, therapeutic, and diagnostic development (Cohen, 2020). In conclusion, AVPs are structurally and functionally versatile owing to its simple primary structure and could serve as the molecular templates for the generation of fast-track therapeutic candidates in the face of COVID-19 or emerging outbreaks posing severe public health threats in the unpredictable future. Given its unique and promising antiviral activity, the potential use of AVPs in clinical treatment and as prophylaxis against COVID-19 should be further explored.

## Author Contributions

AM, YL, C-MF, H-SL, and CL conceived the idea. AM and CL wrote the article. YL, C-MF, and H-SL provided critical comments on the article. AM, YL, C-MF, H-SL, and CL revised the article. All authors contributed to the article and approved the submitted version.

## Funding

The current work was supported by the Fundamental Research Grant Scheme (FRGS), Ministry of Education Malaysia (MOE), grant number FRGS/1/2018/STG05/UNIM/03/1.

## Conflict of Interest

The authors declare that the research was conducted in the absence of any commercial or financial relationships that could be construed as a potential conflict of interest.
